# Identification of Molecular Subtypes and Potential Small-Molecule Drugs for Esophagus Cancer Treatment Based on m^6^A Regulators

**DOI:** 10.1155/2022/5490461

**Published:** 2022-01-13

**Authors:** Jianjun Li, Hongbo Zhu, Qiao Yang, Hua Xiao, Haibiao Wu, Zhe Fang, Wenjun Li, Manbo Cai

**Affiliations:** ^1^Department of Urinary Surgery, The Second Affiliated Hospital, Hengyang Medical School, University of South China, Hengyang City, Hunan Province, 421001, China; ^2^Department of Medical Oncology, The First Affiliated Hospital, Hengyang Medical School, University of South China, Hengyang City, Hunan Province, 421001, China; ^3^Department of Oncology Radiotherapy, The First Affiliated Hospital, Hengyang Medical School, University of South China, Hengyang City, Hunan Province, 421001, China; ^4^Department of Endoscopy Center, The First Affiliated Hospital, Hengyang Medical School, University of South China, Hengyang City, Hunan Province, 421001, China

## Abstract

**Background:**

Esophagus cancer (ESCA) is the sixth most frequent cancer in males, with 5-year overall survival of 15%–25%. RNA modifications function critically in cancer progression, and m^6^A regulators are associated with ESCA prognosis. This study further revealed correlations between m^6^A and ESCA development.

**Methods:**

Univariate Cox regression analysis and consensus clustering were applied to determine molecular subtypes. Functional pathways and gene ontology terms were enriched by gene set enrichment analysis. Protein-protein interaction (PPI) analysis on differentially expressed genes (DEGs) was conducted for hub gene screening. Public drug databases were employed to study the interactions between hub genes and small molecules.

**Results:**

Three molecular subtypes related to ESCA prognosis were determined. Based on multiple analyses among molecular subtypes, 146 DEGs were screened, and a PPT network of 15 hub genes was visualized. Finally, 8 potential small-molecule drugs (BMS-754807, gefitinib, neratinib, zuclopenthixol, puromycin, sulfasalazine, and imatinib) were identified for treating ESCA.

**Conclusions:**

This study applied a new approach to analyzing the relation between m^6^A and ESCA prognosis, providing a reference for exploring potential targets and drugs for ESCA treatment.

## 1. Introduction

Esophagus cancer (ESCA) is the sixth leading cancer with 3.1% incidence and 5.5% mortality worldwide [1]. Males tend to have a higher incidence (4.2%) and mortality (6.8%) than females, mainly due to differences in the smoking and drinking habits between two genders. The major risk factors of ESCA include race, gender, alcohol, tobacco, obesity, gastroesophageal reflux disease (GRED), diet of low fruits and vegetables, and so on [2]. The 5-year overall survival (OS) of the cancer is about 15% to 25%, and poor treatment outcomes are closely associated with late diagnosis and metastasis [3].

In the recent decades, the development of molecular and sequencing technology has deepened the understanding of the genetic causes of ESCA. For example, CCND1, CDK4/CDK6, and MDM2 genes involved in cell cycle are overexpressed in ESCA patients [4]. High expression of epidermal growth factor receptor (EGFR) is associated with worse prognosis and late clinical stage; therefore EGFR expression could serve as a prognostic biomarker [5, 6]. More importantly, epigenetic factors such as DNA or RNA methylation, histone modifications, and loss of genome imprinting show strong correlation with tumor progression [7–9]. These epigenetic alternations can regulate downstream or upstream gene expression through silencing or activating regulatory factors, resulting in aberrant gene expressions associated with tumor development.

RNA modifications in transcripts are the most commonly detected epigenetic alternation. N^6^-methyladenosine (m^6^A) accounts for a majority of RNA modifications under the control of methyltransferases (writers), binding proteins (readers), and demethylases (erasers) [10]. Evidence demonstrated that m^6^A modification is involved in tumorigenesis, tumor proliferation, and migration of various types of cancers such as acute myeloid leukemia [11–14], glioblastoma [15, 16], lung cancer [17, 18], hepatocellular carcinoma [19–21], breast cancer [22], and colorectal cancer [23].

Previous studies also discovered a correlation between m^6^A and ESCA. Nagaki et al. proved that knockdown of ALKBH5 can increase m^6^A modification and delay cell cycle progression of esophageal squamous cell carcinoma (ESCC) cells [24]. ALKBH5 has been identified as m^6^A demethylase that facilitates tumor cell proliferation, and a correlation between ALKBH5 and poor prognosis of ESCC patients has been illustrated [24]. Guo et al. observed that high expression of m^6^A reader HNRNPA2B1 was positively associated with ESCA tumor size and lymphatic metastasis [25]. Knockdown of HNRNPA2B1 inhibits tumor cell progression of ESCA cells, indicating HNRNPA2B1 as an oncogenic protein in ESCA development [25]. In addition, HNRNPC and ALKBH5 have been screened and combined as a prognostic signature for predicting ESCA outcomes [26]. These findings provide potential application of m^6^A regulators in clinical treatment.

As m^6^A modification is strongly correlated with tumor proliferation, invasion, and migration, the present study aimed to exploit novel molecular subtypes of ESCA based on m^6^A regulators (writers, readers, and erasers). Furthermore, hub genes associated with ESCA prognosis and potential small-molecule drugs were screened according to molecular subtypes. This study introduced a new strategy of bioinformatics analysis to explore potential drugs for ESCA treatment.

## 2. Materials and Methods

### 2.1. Data Information

**Figure 1:**
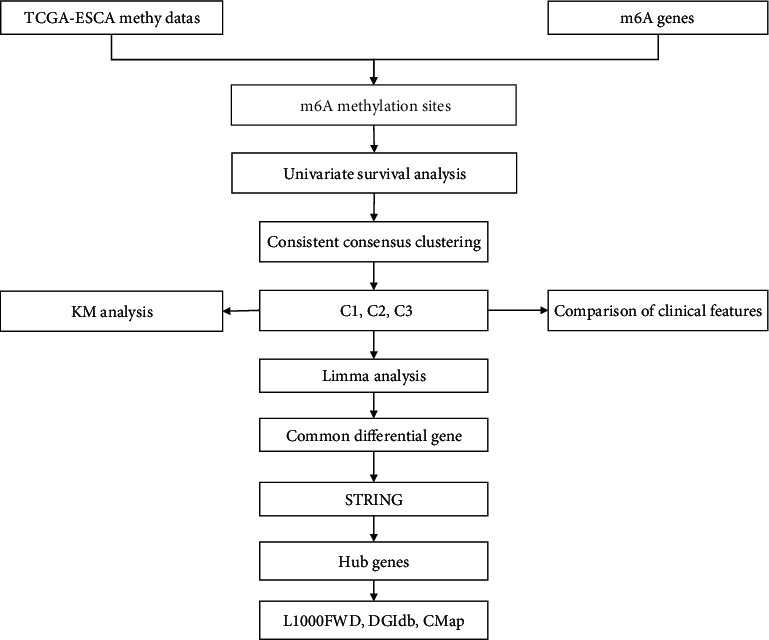
The workflow of developing m^6^A-related molecular subtypes and screening potential small-molecular drugs for treating ESCA.

TCGA-ESCA dataset with the data of gene expression, copy number variation (CNV), single nucleotide variant, methylation, and clinical information was downloaded from The Cancer Genome Atlas (TCGA, https://portal.gdc.cancer.gov/) database. The workflow of defining molecular subtypes of ESCA was shown in Figure 1.

### 2.2. Genes of m^6^A Writers, Erasers, and Readers

Three types of enzymes (writers, erasers, and readers) related to m^6^A were included. Specifically, m^6^A writers were METTL3, METTL14, WTAP, and KIAA1429. m^6^A erasers were FTO and ALKBH5. m^6^A readers were YTHDC1, YTHDC2, YTHDF1, YTHDF2, YTHDF3, HNRNPA2B1, IGF2BP1, IGF2BP2, and IGF2BP3.

### 2.3. Data Preprocessing

RNA-seq and methylation data were further processed. For RNA-seq data, samples without clinical follow-up information, overall survival (OS), and survival status were excluded. Genes with transcripts per million (TPM) < 1 in over half of the samples were excluded. Primary solid tumor samples were included. For methylation data, NA value was completed by the KNN function in impute *R* package, and beta value was converted to *M* value. According to cross-reactive probes and polymorphic CpGs in the Illumina Infinium HumanMethylation450 microarray, CpGs present in the normal samples were excluded. Unstable methylation sites including CpGs in *X* and *Y* chromosomes as well as CpGs in single nucleotide were excluded. Finally, 161 ESCA samples were included (Supplementary Table S1).

### 2.4. Consensus Clustering

R package of ConsensusClusterPlus (v1.48.0) was used to cluster methylation sites related to ESCA prognosis [27] under the parameters of reps = 100, pItem = 0.8, pFeature = 1, distance = “spearman”. D2 algorithm and Euclidean distance were employed for consensus clustering. Cluster numbers *k* from 2 to 10 were chosen, and the optimal clusters were screened by cumulative distribution function (CDF) curve and consensus CDF.

### 2.5. Gene Enrichment Analysis and Function Analysis

Single sample gene set enrichment analysis (ssGSEA) in GSVA *R* package was conducted to calculate the enrichment score of each sample to different functional pathways [28]. WebGestalt (v0.4.3) *R* package was performed to analyze Kyoto Encyclopedia of Genes and Genomes (KEGG) pathways and gene ontology (GO) enrichment for differentially expressed genes (DEGs).

### 2.6. Immune Correlation Analysis

We obtained immune checkpoint genes (BTLA, CD200, CD244, LAG3, IDO1, IDO2, PDCD1, CTLA4, PDCD1LG2, TNFRSF8, CD40, TNFSF18, CD86, and CD44) from previous studies to analyze the expression differences of these genes in various molecular subtypes. In addition, we evaluated 28 immune infiltrating cell components by ssGSEA method [29]. To analyze the differences of immune infiltrating cell components in different subtypes, we further evaluated the immune infiltrating score in the sample by using *R* software package estimate [30], analyzed the differences of immune infiltrating in different subtypes, and evaluated the potential benefits of immunotherapy of different subtypes in imvigor210 [31] by using *R* software package submap [32].

### 2.7. Protein-Protein Interaction (PPI) Analysis

STRING (https://string-db.org/) is a database to explore the interaction among known and unknown proteins, including abundant data from current researches, other databases, and data by predicted bioinformatics [33, 34]. The protein interactions of DEGs were analyzed by STRING. PPI result was visualized by Cytoscape (v3.7.2) and further analyzed by cytoHubba to screen hub genes [35–37].

### 2.8. Databases of Small-Molecule Drugs

Databases of L1000 fireworks display (L1000FWD, https://maayanlab.cloud/L1000FWD/) [38], Drug-Gene Interaction database (DGIdb, https://dgidb.org/) [39, 40], and The Connectivity Map (CMap, https://clue.io/) [41, 42] were included for screening small molecules interacting with hub genes. L1000FWD includes over 16000 small molecules and gene expression data from tumor cell lines of 1000 drugs. DGIdb stores over 10000 drugs and 15000 interactions between drugs and genes. CMap contains over 19000 small molecules corresponding to 25200 biological entities. The function of small molecules associated with hub genes came from the National Library of Medicine (PubChem, https://pubchem.ncbi.nlm.nih.gov/#query=).

## 3. Results

### 3.1. Consensus Clustering of Methylation Sites on 15 m^6^A-Related Genes

**Figure 2:**
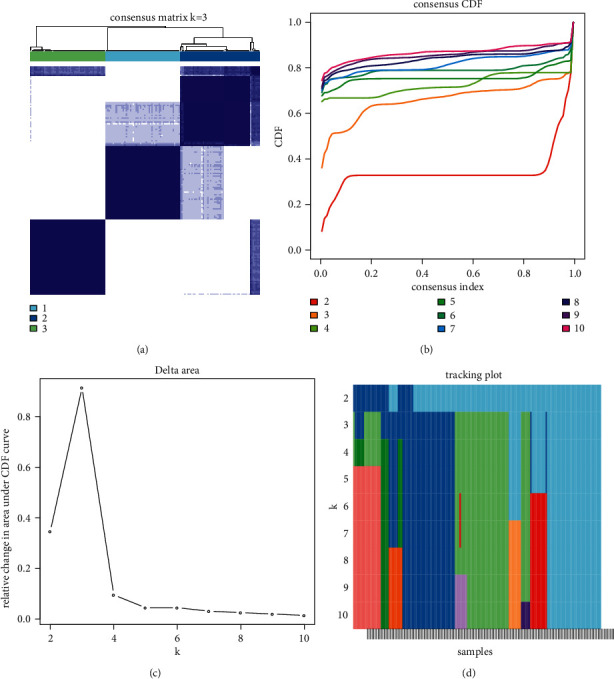
Consensus clustering of 161 ESCA samples based on m^6^A methylation sites. (a) A consensus matrix when *k* = 3 where k represents cluster number. (b) Consensus CDF when *k* = 2 to 10. (c) The relative change in area under CDF curve when *k* = 2 to 10. (d) Tracking plot of samples when *k* = 2 to 10.

To develop a molecular subtyping system based on m^6^A methylation sites, association between m^6^A methylation sites and ESCA prognosis was analyzed. Coxph function in *R* package survival was used to perform univariate Cox regression analysis between 221 methylation sites and OS, survival status. 9 methylation sites associated with prognosis were screened (*p* < 0.05, Supplementary Table S2). Then 161 ESCA samples were clustered based on the 9 methylation sites with consensus clustering in ConsensusClusterPlus *R* package. As shown in Figure 2, when cluster number *k* = 3, CDF did not show great change; meanwhile, the relative change in area under CDF curve was the maximum, suggesting that *k* = 3 was the optimal. Therefore, under *k* = 3, 161 ESCA samples were clustered into three subtypes of C1, C2, and C3.

**Figure 3:**
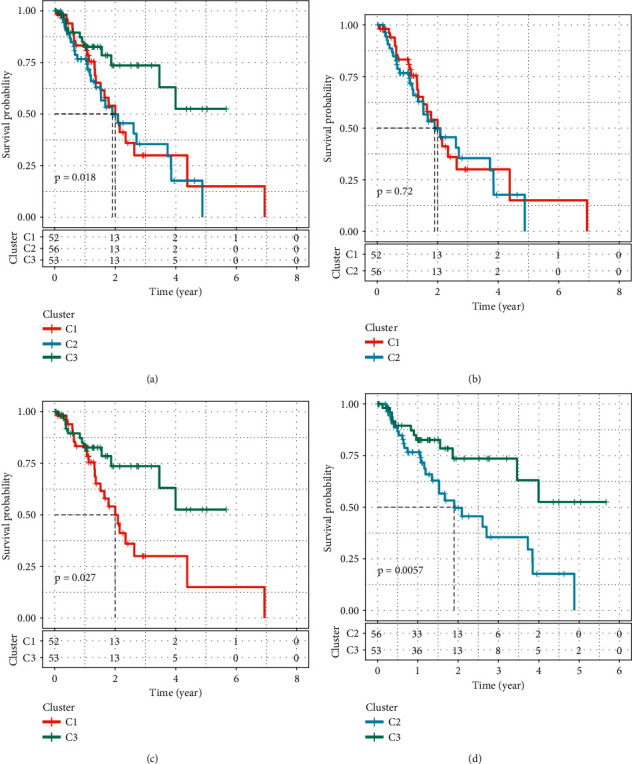
Kaplan-Meier survival curve of C1, C2, and C3 subtypes. (a) Survival analysis among three subtypes (*p*=0.018). (b) Survival analysis between C1 and C2 subtypes (*p*=0.72). (c) Survival analysis between C1 and C3 subtypes (*p*=0.027). (d) Survival analysis between C1 and C3 subtypes (*p*=0.0057). Log-rank test was performed.

Survival analysis manifested significant OS difference in the three subtypes, with the most favorable prognosis detected in C3 subtype (*p*=0.018, Figure 3). However, no difference of OS was observed between C1 and C2 subtypes. Gene mutation analysis showed that the top mutated gene was TP53 and that C3 subtype had the least mutations of the top 20 mutated genes among three subtypes (Supplementary Figure S1). Such results suggested a relation between gene mutations and prognosis.

### 3.2. The Relation between the Three Subtypes and Clinical Features

**Figure 4:**
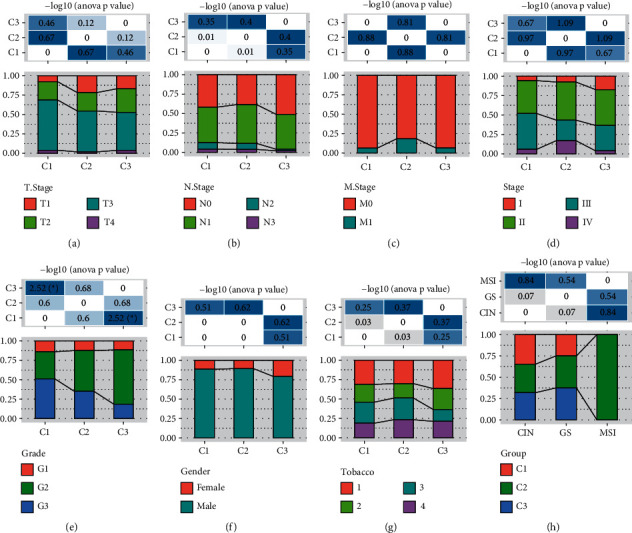
The relation between three subtypes and clinical features, including T stage (a), N stage (b), M stage (c), stages I to IV (d), grade (e), gender (f), tobacco (g), and TCGA molecular subtypes (h). ANOVA was performed. ^*∗*^*p* < 0.05.

We next assessed the relation between subtypes and clinical features, including *T* stage, *N* stage, *M* stage, stages I to IV, grade, and risk factors of gender and smoking history. Although only the distribution of *G* stage (*G*1, *G*2, and *G*3) showed a close relation with the three subtypes (*p* < 0.05), there was a modest tendency indicating that C3 subtype had the lower proportion of stages with more invasive features than C1 and C2 subtypes (Figures 4(a)–4(e)). For risk factors, males accounted for a significantly higher proportion than female due to a higher smoking in males, and the female proportion was the highest in C3 subtype (Figure 4(f)). In addition, the number of nonsmokers was more in C3 subtype (tobacco = 1) than C1 and C2 subtypes; however, no significant difference was detected among tobacco groups (Figure 4(g)). We compared the three molecular subtypes with the previously reported three molecular subtypes (CIN, GS, and MSI) [43]. We observed that MSI subtypes are mainly related to C2 (Figure 4(h)). For example, the distribution of C1, C2, and C3 in CIN and GS subtypes is similar, suggesting that the new three molecular subtypes can be used as a supplement to the previously reported molecular subtypes.

### 3.3. The Enrichment of Metabolism Pathways in the Three Subtypes

**Figure 5:**
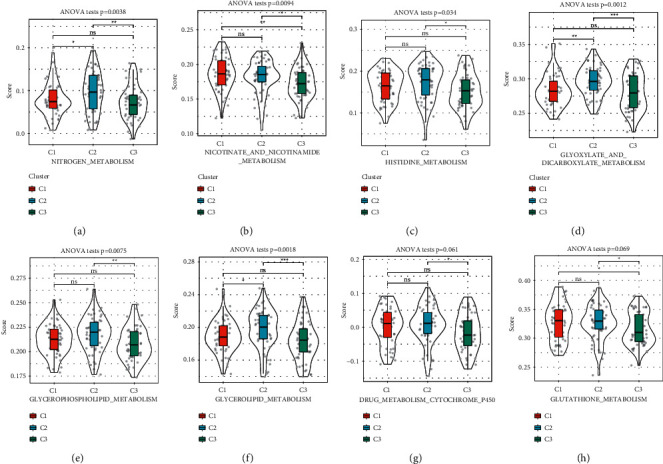
The relation between the three subtypes and metabolism pathways, including nitrogen metabolism (a), nicotinate and nicotinamide metabolism (b), histidine metabolism (c), glyoxylate and dicarboxylate metabolism (d), glycerophospholipid metabolism (e), glycerolipid metabolism (f), drug metabolism cytochrome p450 (g), and glutathione metabolism (h). ANOVA was performed. ns, no significance. ^*∗*^*p* < 0.05, ^*∗∗*^*p* < 0.01, ^*∗∗∗*^*p* < 0.001.

Compared with normal cells, tumor cells are more active in acquiring energy through metabolism pathways to promote cell proliferation and migration. Therefore, we speculated that the activity of tumor cells in metabolism pathways could indicate the condition of cancer patients' prognosis. To examine whether there was an association between subtypes and metabolism pathways, ssGSEA was conducted to calculate the enrichment score of each sample. Eight major metabolism pathways, including nitrogen metabolism, nicotinate and nicotinamide metabolism, histidine metabolism, glyoxylate and dicarboxylate metabolism, glycerophospholipid metabolism, glycerolipid metabolism, drug metabolism cytochrome p450, and glutathione metabolism, were analyzed. The result exhibited that C3 subtype had the lowest enrichment score in these pathways, suggesting that C3 subtype with favorable prognosis was relatively inactive in metabolism pathways (Figure 5).

### 3.4. Immune Correlation of Different Molecular Subtypes

**Figure 6:**
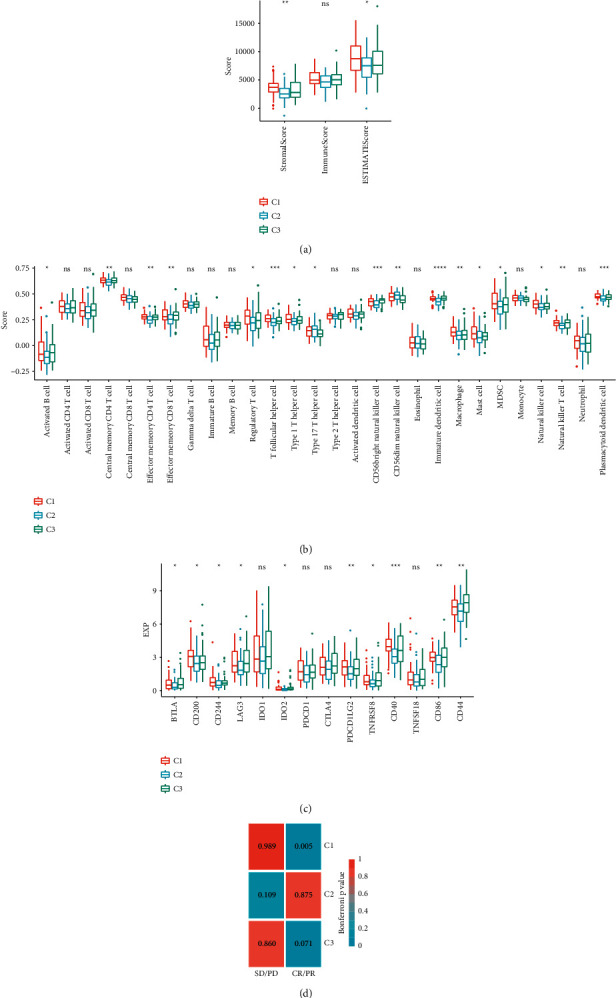
Immune correlation of three molecular subtypes. (a) Different distribution of immune infiltration of different molecular subtypes. (b) The distribution of immune cell infiltration in different molecular subtypes was different. (c) The expression and distribution of immune checkpoint genes of different molecular subtypes were different. (d) Correlation of immunotherapeutic response of PD-L1 with different molecular subtypes. Analysis of variance was used to test the difference between multiple groups of samples.

Immunotherapy is a promising clinical treatment method. In order to characterize the potential benefits of immunotherapy of different molecular subtypes, we first compared the differences of immune infiltration in the immune microenvironment of the three molecular subtypes. It can be observed that C1 subtype has higher matrix components and higher tumor purity (Figure 6(a)). We also observed the differences of multiple immune cell infiltration in patients with three molecular subtypes (Figure 6(b)). For example, C3 subtype has higher effector memory CD8 T cell and activated B cell, and C1 subtype has the highest regulatory T cell. These results show that the three molecular subtypes have different immune microenvironment characteristics. Further, we analyzed the expression differences of immune checkpoint genes in the three molecular subtypes and observed that 10 (71%) immune checkpoint genes had significant expression differences (Figure 6(c)), of which CD40 had the most significant expression difference. In addition, we also observed that C1 subtype was significantly correlated with CR/PR (Figure 6(d)). This suggests that C1 subtype may benefit from immunotherapy of PD-L1.

Identification of differentially expressed genes among the three subtypes and functional analysis were done.

**Figure 7:**
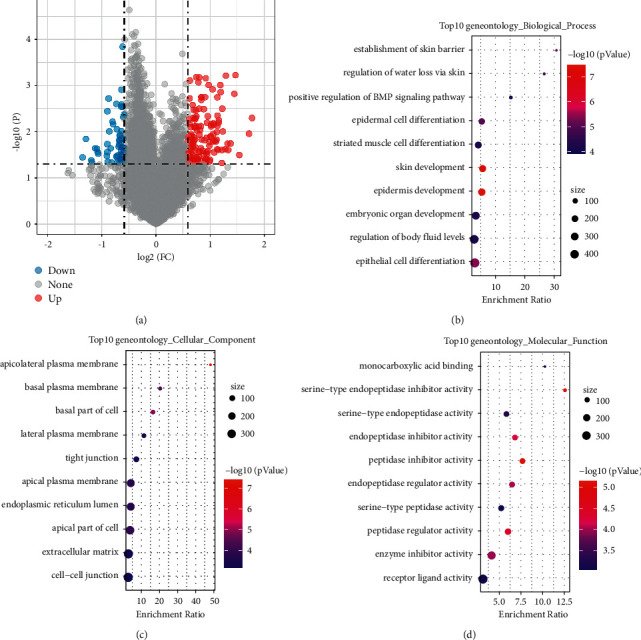
The volcano plot of DEGs between C1 and C3 subtypes. Blue represents downregulated genes and red represents upregulated genes. FC, fold change (a). The top 10 annotated terms of biological process (b), cellular component (c), and molecular function (d) between C1 and C3 subtypes. Dot size represents the gene numbers. The annotated terms were displayed in vertical axis and the enrichment ratio of each term was displayed in horizontal axis.

As no difference of OS was found between C1 and C2 subtypes, and C3 subtype had the optimal prognosis, we also analyzed the DEGs between C1 and C3 and between C2 and C3 subtypes. Between C1 and C3 subtypes, 193 DEGs (132 upregulated genes and 61 downregulated genes) were identified under conditions of *p* < 0.05 and |fold change (FC)| > 1.5 using Limma *R* package (Figure 7(a)). Then 193 DEGs were further assessed with GO function analysis and KEGG pathways using WebGestalt *R* package. GO analysis showed that 432 terms of biological process, 27 terms of cellular component, and 41 terms of molecular function were annotated with significant differences between C1 and C3 subtypes (*p* < 0.05). The top 10 enriched terms of biological process, cellular component, and molecular function were displayed (Figures 7(b)–7(d)). However, no KEGG pathways with significant difference between C1 and C3 subtypes were found. Moreover, between C2 and C3 subtypes, we identified 1673 DEGs incorporating 685 upregulated and 988 downregulated genes (Supplementary Figure S2) and annotated 35 KEGG pathways, 1181 terms of biological process, 132 terms of cellular component, and 153 terms of molecular function. The top 10 enriched terms were shown in Supplementary Figure S3. Among these terms, epidermal cell differentiation, striated muscle cell differentiation, skin development, epidermis development, and epithelial cell differentiation were all annotated in the top 10 biological processes between C1 and C3 and between C2 and C3 (Figure 7 and Supplementary Figure S3).

### 3.5. Construction of PPI Networks and Hub Gene Identification

**Figure 8:**
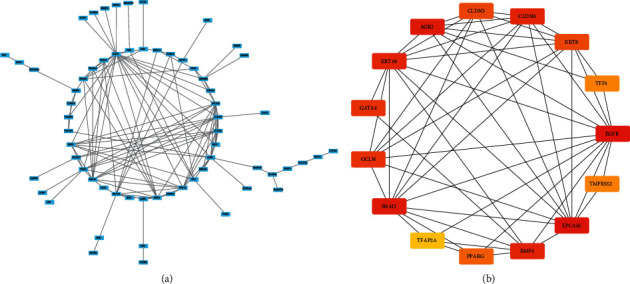
PPI analysis of 146 upregulated and downregulated genes. (a) A PPI network of 146 DEGs constructed by STRING. (b) A PPI network of 15 hub genes screened by Cytoscape and cytoHubba. Black line represents the interactions between proteins.

Next, mutually upregulated and downregulated DEGs between C1 and C3 and between C2 and C3 subtypes were examined. 146 mutual DEGs including 97 upregulated and 49 downregulated ones were identified for constructing PPI networks (Figure 8(a)). The bioinformatics tools in STRING database were applied to assess the interactions among 146 proteins of DEGs. Subsequently, Cytoscape was applied to visualize the PPI network and cytoHubba was performed to screen hub genes from the network (Figure 8(b)). Finally, the following top 15 hub genes were identified: OCLN, TFF1, BMP4, KRT18, CLDN3, CLDN4, KRT8, TFAP2A, PPARG, AGR2, GATA4, EPCAM, SNAI2, EGFR, and TMPRSS2. We further evaluated the expression differences of these 15 genes in cancer and adjacent tumors. We observed that GATA4, AGR2, and PPARG were significantly underexpressed in tumor samples (Supplementary Figure S4A). We further evaluated the methylation level of CpG sites in the promoter region of these 15 genes in each sample. It can be observed that there is a higher methylation level in cancer samples as a whole, in particular, GATA4 and TFAP2A (Supplementary Figure S4B). We used ssGSEA to evaluate the enrichment scores of six important immune pathways and further analyzed the correlation between these 15 genes and these immune pathways. It was observed that there was a higher correlation between these genes and weak correlation with immune pathways, among which EPCAM was the most correlated with immune pathways (Supplementary Figure S4(c)).

### 3.6. Screening of Small Molecules Related to Hub Genes

**Figure 9:**
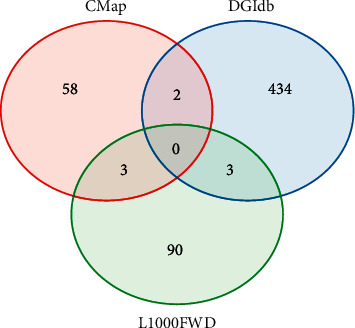
Screening of small molecules related to hub genes from L1000FWD, DGIdb, and CMap databases.

The 15 hub genes were screened from DEGs between C1 and C3, C2 and C3 were considered to be closely related to ESCA prognosis, and this also suggested that these genes could be the targets for ESCA treatment. Therefore, we introduced three databases of L1000FWD, DGIdb, and CMap with abundant data of the interactions between small-molecule drugs and genes. If one drug is negatively associated with expression of one gene related to ESCA, the drug could be considered as a potential drug for ESCA treatment. Within three databases, we screened a total of 598 small molecules having interactions with hub genes, including 96 from L1000FWD, 439 from DGIdb, and 63 from CMap. By overlapping these small molecules in three databases, we observed 3 small molecules (BMS-754807, gefitinib, and neratinib) were overlapped between L1000FWD and DGIdb, 3 small molecules (zuclopenthixol, puromycin, and naringenin) were overlapped between L1000FWD and CMap, 2 small molecules (sulfasalazine and imatinib) were overlapped between DGIdb and CMap (Figure 9). Among these 8 small molecules, BMS-754807, gefitinib, neratinib, and imatinib have antitumor activity. Zuclopenthixol, as a Dopamine receptor antagonist, is a drug for treating schizophrenia. Puromycin is an aminoglycoside antibiotic, and sulfasalazine is a nonsteroid anti-inflammatory drug. These drugs may specifically target hub genes and take function in suppressing tumor cell proliferation and invasion, although further experiment and analysis are needed for illustrating their function and mechanism in antitumor activity.

## 4. Discussion

A number of epigenetic studies on ESCA have revealed the significance of epigenetic regulation on ESCA development; however, the role of m^6^A modification on ESCA has not been systematically studied. Only several studies have found that some m^6^A regulators, such as ALKBH5, HNRNPA2B1, and HNRNPC, have strong relation with ESCA prognosis [24–26]. Inspired from the previous researches, we focused on analyzing a total of 15 m^6^A regulators and identified three new molecular subtypes associated with clinical features and ESCA prognosis. Furthermore, we constructed a PPI network based on DEGs screened from the three subtypes and determined 15 prognosis-related hub genes from the PPI network.

Some of the 15 hub genes have been reported to be associated with tumor progression of ESCA. For example, TFF1 encodes a mucosa protector factor, and it is silenced in the early stage of ESCA development resulting from high methylation of TFF1 promoter [44]. BMP4 and EPCAM are involved in inducing epithelial-mesenchymal transition (EMT) and promoting tumor cell migration of ESCA [45–47]. Low expression of CLDN4 is indicative of a poor prognosis of ESCC [48]. High expression of TFAP2A is correlated with favorable OS of ESCC patients [49]. EGFR is highly expressed in ESCA and some other cancer types; moreover, it is seen as a promising target for inhibiting tumor aggression [50]. Although some hub genes have not been found to be correlated with ESCA development, their relations with other cancer types have been previously demonstrated.

Using small-molecule databases, 8 potential drugs closely interacting with the 15 hub genes were identified. These drugs negatively associated with expression of the hub genes can be considered as potential drugs for treating ESCA. Among the 8 drugs, gefitinib, neratinib, and imatinib have been commercialized for clinical treatment of specific cancers. Gefitinib is an EGFR tyrosine kinase inhibitor that can hinder tumor cell proliferation and angiogenesis and has been commercially applied in treating non-small-cell lung cancer [51]. Clinical trials of gefitinib in advanced ESCA patients demonstrated a partial response and stable disease rate of between 24% and 39%, showing a relatively positive effect [52–54]. Neratinib is a tyrosine kinase inhibitor targeting HER1, HER2, and HER4 and can effectively improve disease-free survival of HER2-positive breast cancer patients given with chemotherapy and trastuzumab [55, 56]. Imatinib, a tyrosine kinase inhibitor targeting Bcr-Abl tyrosine kinase, could suppress disease progression and extend overall survival of chronic myeloid leukemia and gastrointestinal stromal tumors [57, 58].

BMS-754807 has not been used to treat cancers; however, evidence suggested a promising application of it in clinical practice. BMS-754807 is an inhibitor of targeting insulin-like growth factor-1 receptor/insulin receptor (IGF-1R/IR) signaling pathway, which has been proven to be effective in suppressing tumor cell proliferation of xenograft tumor models of several cancer types [59–61]. Study found that sulfasalazine could enhance cisplatin-induced cytotoxic effects on advanced gastric cancer and bladder cancer [62, 63]. The remaining two drugs zuclopenthixol and puromycin have not been reported to be related to cancer therapy, but they still may have the potential to target hub genes related to ESCA prognosis, according to our analysis.

This study did not differentiate two molecular types of esophagus cancer (squamous cell carcinoma and adenocarcinoma), which may affect the results of molecular subtypes to some extent. In addition, further study on these hub genes and small molecules are needed to demonstrate their functions in clinical practice. Importantly, this study applied a new approach to analyzing the relation between m^6^A and ESCA prognosis and provided a valuable reference to explore potential targets and drugs for ESCA treatment.

## 5. Conclusions

In conclusion, this study determined three molecular subtypes of ESCA based on m^6^A regulators and identified 8 potential small-molecule drugs closely interacting with hub genes through integrative analysis. The new molecular subtypes were effective in classifying ESCA patients into low-risk and high-risk groups. The 15 hub genes screened from DEGs among three subtypes can be potential targets for treating ESCA. The 8 small-molecule drugs closely interacting with the hub genes may be promising drugs for ESCA patients.

## Data Availability

The data used to support the findings of this study are included within the article.

## Abbreviations

CDF: Cumulative distribution function
CMap: The connectivity map
CNV: Copy number variation
DEGs: Differentially expressed genes
DGIdb: Drug-gene interaction database
EGFR: Epidermal growth factor receptor
ESCA: Esophagus cancer
ESCC: Esophageal squamous cell carcinoma
FC: Fold change
GO: Gene ontology
GRED: Gastroesophageal reflux disease
IGF-1R/IR: Insulin-like growth factor-1 receptor/insulin receptor
KEGG: Kyoto encyclopedia of genes and genomes
L1000FWD: L1000 fireworks display
m^6^A: N^6^-methyladenosine
OS: Overall survival
PPI: Protein-protein interaction
ssGSEA: Single sample gene set enrichment analysis
TCGA: The cancer genome atlas
TPM: Transcripts per million.

## Supplementary Materials

Supplementary Materials
*Supplementary Figure S1.* The mutation patterns of top 20 mutated genes in C1 (a), C2 (b), and C3 (c) subtypes. *Supplementary Figure S2.* The volcano plot of DEGs between C2 and C3 subtypes. Blue represents downregulated genes and red represents upregulated genes. FC, fold change. *Supplementary Figure S3.* The top 10 annotated terms of biological processes (a), cellular component (b), molecular function (c), and KEGG pathways (D) between C2 and C3 subtypes. Dot size represents the gene numbers. The annotated terms were displayed in vertical axis and the enrichment ratio of each term was displayed in horizontal axis. *Supplementary Figure S4*. a: Differential expression of 15 genes in cancer and adjacent tissues; b: Heatmap of methylation distribution of promoter region methylation sites of 15 genes in various types of samples; c: Correlation heatmap of 15 genes and 6 immune related pathways. *Supplementary Table S1.* The clinical information of 161 ESCA samples. *Supplementary Table S2.* 9 m^6^A methylation sites screened by univariate Cox regression analysis.
