# Supplementary material for: Identification of Molecular Subtypes and Potential Small-Molecule Drugs for Esophagus Cancer Treatment Based on m6A Regulators
Source: J Oncol. 2022 Jan 13;2022:5490461. doi: 10.1155/2022/5490461 (PMC8776445; doi:10.1155/2022/5490461)
Supplement: Supplementary Materials — Supplementary Figure S1. The mutation patterns of top 20 mutated genes in C1 (a), C2 (b), and C3 (c) subtypes. Supplementary Figure S2. The volcano plot of DEGs between C2 and C3 subtypes. Blue represents downregulated genes and red represents upregulated genes. FC, fold change. Supplementary Figure S3. The top 10 annotated terms of biological processes (a), cellular component (b), molecular function (c), and KEGG pathways (D) between C2 and C3 subtypes. Dot size represents the gene numbers. The annotated terms were displayed in vertical axis and the enrichment ratio of each term was displayed in horizontal axis. Supplementary Figure S4. a: Differential expression of 15 genes in cancer and adjacent tissues; b: Heatmap of methylation distribution of promoter region methylation sites of 15 genes in various types of samples; c: Correlation heatmap of 15 genes and 6 immune related pathways. Supplementary Table S1. The clinical information of 161 ESCA samples. Supplementary Table S2. 9 m6A methylation sites screened by univariate Cox regression analysis. [file 5490461.f1.zip › 5490461.f1/Supplementary Figure S1.pdf]

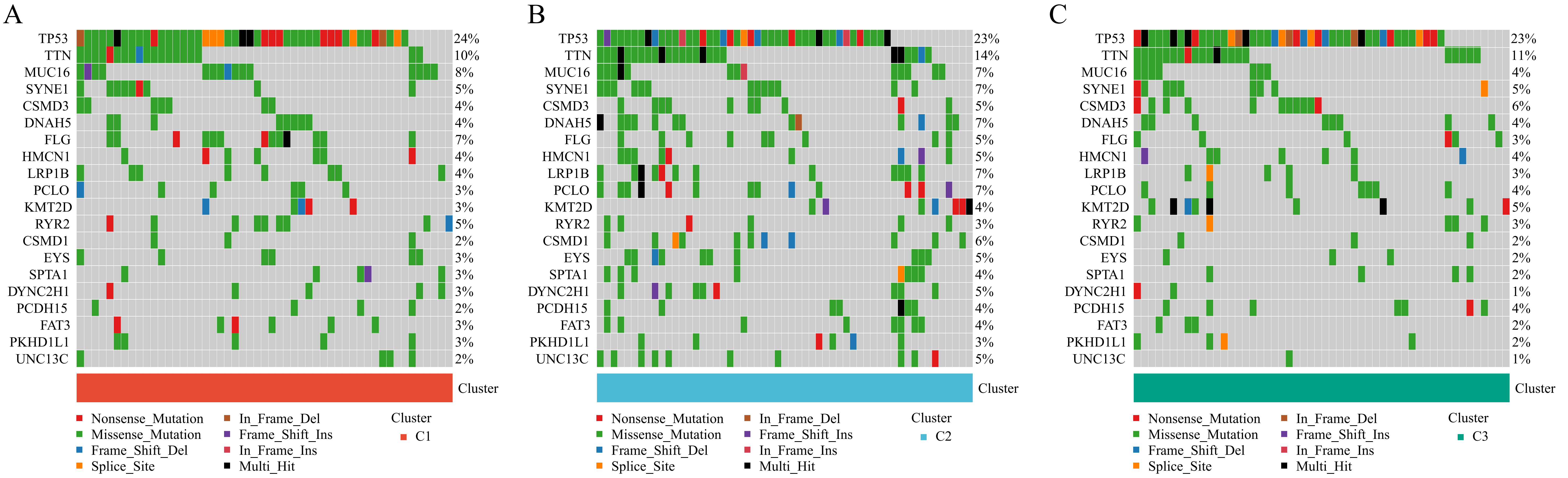
A
TP53
TTN
MUC16
SYNE1
CSMD3
DNAH5
FLG
HMCN1
LRP1B
PCLO
KMT2D
RYR2
CSMD1
EYS
SPTA1
DYNC2H1
PCDH15
FAT3
PKHD1L1
UNC13C
24%
10%
8%
5%
4%
4%
7%
4%
4%
3%
3%
5%
2%
3%
3%
3%
2%
3%
3%
2%
Cluster
Nonsense_Mutation
Missense_Mutation
Frame_Shift_Del
Splice_Site
In_Frame_Del
Frame_Shift_Ins
In_Frame_Ins
Multi_Hit
Cluster
C1
B
TP53
TTN
MUC16
SYNE1
CSMD3
DNAH5
FLG
HMCN1
LRP1B
PCLO
KMT2D
RYR2
CSMD1
EYS
SPTA1
DYNC2H1
PCDH15
FAT3
PKHD1L1
UNC13C
23%
14%
7%
7%
5%
7%
5%
5%
7%
7%
4%
3%
6%
5%
4%
5%
4%
4%
3%
5%
Cluster
Nonsense_Mutation
Missense_Mutation
Frame_Shift_Del
Splice_Site
In_Frame_Del
Frame_Shift_Ins
In_Frame_Ins
Multi_Hit
Cluster
C2
C
TP53
TTN
MUC16
SYNE1
CSMD3
DNAH5
FLG
HMCN1
LRP1B
PCLO
KMT2D
RYR2
CSMD1
EYS
SPTA1
DYNC2H1
PCDH15
FAT3
PKHD1L1
UNC13C
23%
11%
4%
5%
6%
4%
3%
4%
3%
4%
5%
3%
2%
2%
2%
1%
4%
2%
2%
1%
Cluster
Nonsense_Mutation
Missense_Mutation
Frame_Shift_Del
Splice_Site
In_Frame_Del
Frame_Shift_Ins
In_Frame_Ins
Multi_Hit
Cluster
C3
