# Supplementary material for: Identification of Molecular Subtypes and Potential Small-Molecule Drugs for Esophagus Cancer Treatment Based on m6A Regulators
Source: J Oncol. 2022 Jan 13;2022:5490461. doi: 10.1155/2022/5490461 (PMC8776445; doi:10.1155/2022/5490461)
Supplement: Supplementary Materials — Supplementary Figure S1. The mutation patterns of top 20 mutated genes in C1 (a), C2 (b), and C3 (c) subtypes. Supplementary Figure S2. The volcano plot of DEGs between C2 and C3 subtypes. Blue represents downregulated genes and red represents upregulated genes. FC, fold change. Supplementary Figure S3. The top 10 annotated terms of biological processes (a), cellular component (b), molecular function (c), and KEGG pathways (D) between C2 and C3 subtypes. Dot size represents the gene numbers. The annotated terms were displayed in vertical axis and the enrichment ratio of each term was displayed in horizontal axis. Supplementary Figure S4. a: Differential expression of 15 genes in cancer and adjacent tissues; b: Heatmap of methylation distribution of promoter region methylation sites of 15 genes in various types of samples; c: Correlation heatmap of 15 genes and 6 immune related pathways. Supplementary Table S1. The clinical information of 161 ESCA samples. Supplementary Table S2. 9 m6A methylation sites screened by univariate Cox regression analysis. [file 5490461.f1.zip › 5490461.f1/Supplementary Figure S3.pdf]

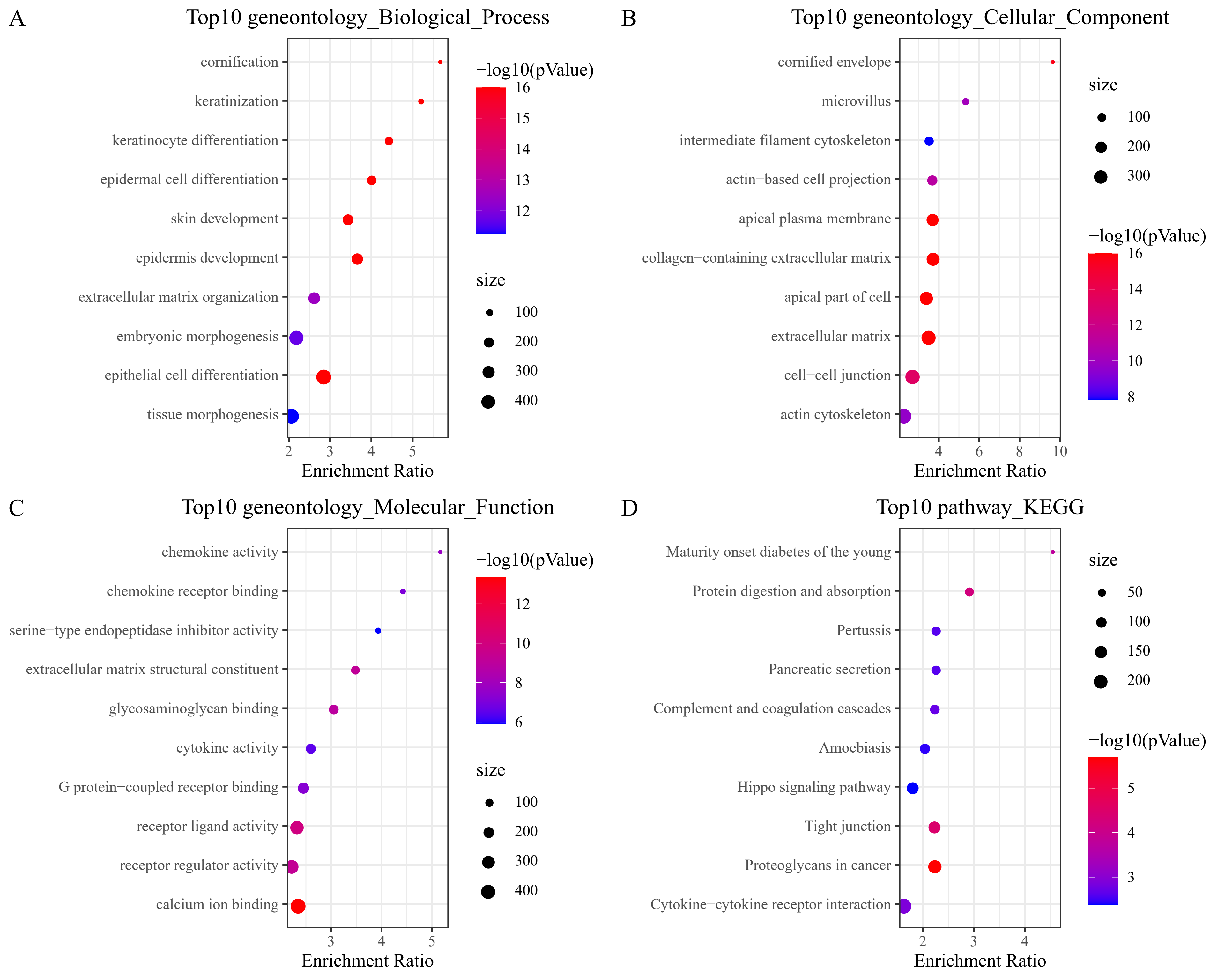
A
Top10 geneontology_Biological_Process
cornification
keratinization
keratinocyte differentiation
epidermal cell differentiation
skin development
epidermis development
extracellular matrix organization
embryonic morphogenesis
epithelial cell differentiation
tissue morphogenesis
Enrichment Ratio
−log10(pValue)
size
B
Top10 geneontology_Cellular_Component
cornified envelope
microvillus
intermediate filament cytoskeleton
actin−based cell projection
apical plasma membrane
collagen−containing extracellular matrix
apical part of cell
extracellular matrix
cell−cell junction
actin cytoskeleton
Enrichment Ratio
size
−log10(pValue)
C
Top10 geneontology_Molecular_Function
chemokine activity
chemokine receptor binding
serine−type endopeptidase inhibitor activity
extracellular matrix structural constituent
glycosaminoglycan binding
cytokine activity
G protein−coupled receptor binding
receptor ligand activity
receptor regulator activity
calcium ion binding
Enrichment Ratio
−log10(pValue)
size
D
Top10 pathway_KEGG
Maturity onset diabetes of the young
Protein digestion and absorption
Pertussis
Pancreatic secretion
Complement and coagulation cascades
Amoebiasis
Hippo signaling pathway
Tight junction
Proteoglycans in cancer
Cytokine−cytokine receptor interaction
Enrichment Ratio
size
−log10(pValue)
