# Supplementary material for: Identification of Molecular Subtypes and Potential Small-Molecule Drugs for Esophagus Cancer Treatment Based on m6A Regulators
Source: J Oncol. 2022 Jan 13;2022:5490461. doi: 10.1155/2022/5490461 (PMC8776445; doi:10.1155/2022/5490461)
Supplement: Supplementary Materials — Supplementary Figure S1. The mutation patterns of top 20 mutated genes in C1 (a), C2 (b), and C3 (c) subtypes. Supplementary Figure S2. The volcano plot of DEGs between C2 and C3 subtypes. Blue represents downregulated genes and red represents upregulated genes. FC, fold change. Supplementary Figure S3. The top 10 annotated terms of biological processes (a), cellular component (b), molecular function (c), and KEGG pathways (D) between C2 and C3 subtypes. Dot size represents the gene numbers. The annotated terms were displayed in vertical axis and the enrichment ratio of each term was displayed in horizontal axis. Supplementary Figure S4. a: Differential expression of 15 genes in cancer and adjacent tissues; b: Heatmap of methylation distribution of promoter region methylation sites of 15 genes in various types of samples; c: Correlation heatmap of 15 genes and 6 immune related pathways. Supplementary Table S1. The clinical information of 161 ESCA samples. Supplementary Table S2. 9 m6A methylation sites screened by univariate Cox regression analysis. [file 5490461.f1.zip › 5490461.f1/Supplementary materials.docx]

## Supplementary materials

Supplementary Figure S1. The mutation patterns of top 20 mutated genes in C1 (A), C2 (B) and C3 (C) subtypes.

Supplementary Figure S2. The volcano plot of DEGs between C2 and C3 subtypes. Blue represents down-regulated genes and red represents up-regulated genes. FC, fold change.

Supplementary Figure S3. The top 10 annotated terms of biological processes (A), cellular component (B), molecular function (C) and KEGG pathways (D) between C2 and C3 subtypes. Dot size represents the gene numbers. The annotated terms were displayed in vertical axis and the enrichment ratio of each term was displayed in horizontal axis.

Supplementary Figure S4. A: Differential expression of 15 genes in cancer and adjacent tissues; B: Heatmap of methylation distribution of promoter region methylation sites of 15 genes in various types of samples; C: Correlation heatmap of 15 genes and 6 immune related pathways.

Supplementary Table S1. The clinical information of 161 ESCA samples.

Supplementary Table S2. 9 m^6^A methylation sites screened by univariate Cox regression analysis.
